# Supplementary material for: Single cell mapping identifies a distinct platelet-phenotype in psoriatic type III inflammation
Source: Nat Commun. 2025 Dec 5;16:10881. doi: 10.1038/s41467-025-65894-7 (PMC12680767; doi:10.1038/s41467-025-65894-7)
Supplement: Supplementary file 1 — Supplementary Information [file 41467_2025_65894_MOESM1_ESM.pdf]

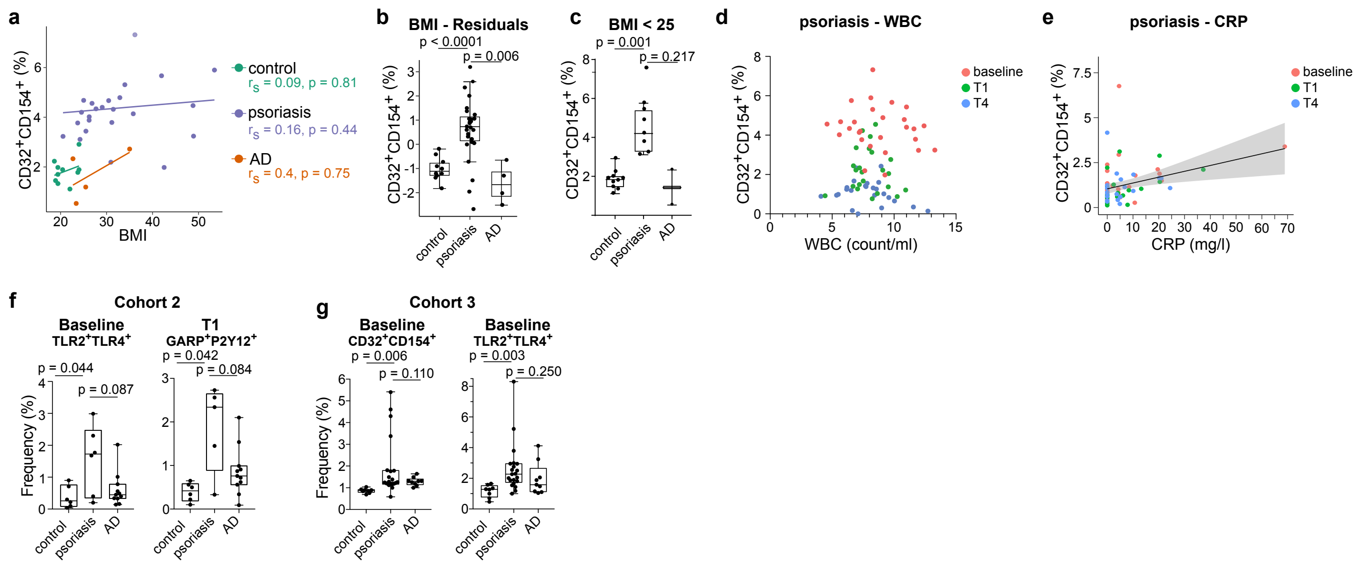

Suppl. Fig. 1: Correction for body mass index and data driven analysis in two additional validation cohorts corroborates distinct type-III specific phenotype of platelets.

a. Spearman correlation analysis between body mass index (BMI) and CD32+CD154+ frequency at baseline within individual groups. In the overall cohort, a significant positive correlation was observed (rs = 0.56, p = 0.00016). However, no significant correlations were found within the individual groups: Control (rs = 0.09, p = 0.808), atopic dermatitis (AD) (rs = 0.40, p = 0.75), and psoriasis (rs = 0.16, p = 0.435). **b.** Visualized residuals of a fitted a linear model (CD32+CD154+ ~ BMI) using boxplots stratified by group. **c.** Quantification of CD32+CD154+ frequencies in BMI matched cohort 1 at baseline, only including individuals with BMI < 25 (n = 10 controls, n = 7 psoriasis patients, n = 2 AD patients,). **d.** Spearman correlation of CD32+CD154+ platelet frequencies with peripheral white blood count (WBC) and c-reactive protein (CRP) in (**e**). Data in **a-e** represents analysis of cohort 1, which was processed, measured and analyzed in one batch (n = 10 controls, n = 27 psoriasis patients, n = 4 AD patients) **f.** Quantification of indicated clusters at indicated timepoints across healthy controls (control, n = 6), psoriasis patients (psoriasis, n = 6) and atopic dermatitis patients (AD, n = 10) in cohort 2, processed, measured and analyzed in a second separate batch. **g.** Quantification of indicated clusters at indicated timepoints across healthy controls (control, n = 8), psoriasis patients (psoriasis, n = 21) and atopic dermatitis patients (AD, n = 9) in cohort 3, processed, measured and analyzed in a third separate batch. Statistical analysis was carried out using one-sided unpaired student's t tests. Source data are provided in a source data file. For all depicted box plots in this figure, the horizontal line within each box indicates the median, while the lower and upper box edges denote the first and third quartiles, respectively. Whiskers extend to 1.5 times the interquartile range, and dots represent individual data points.

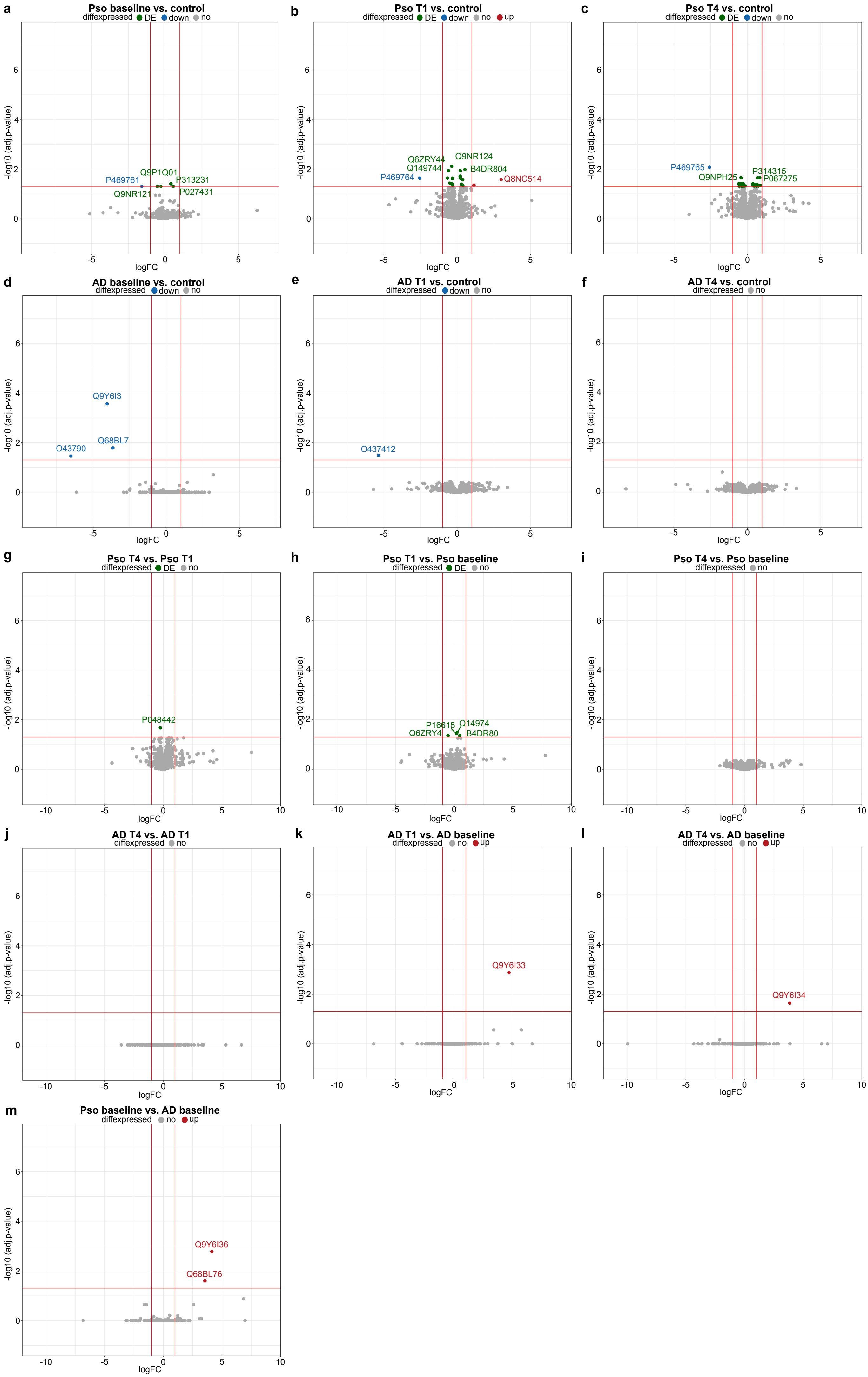
a
Pso baseline vs. control
diffexpressed DE down no
P469761
Q9P1Q01
P313231
Q9NR121
P027431
b
Pso T1 vs. control
diffexpressed DE down no up
Q6ZRY44
Q9NR124
Q149744
B4DR804
P469764
Q8NC514
c
Pso T4 vs. control
diffexpressed DE down no
P469765
P314315
Q9NPH25
P067275
d
AD baseline vs. control
diffexpressed down no
Q9Y6I3
Q68BL7
O43790
e
AD T1 vs. control
diffexpressed down no
O437412
f
AD T4 vs. control
diffexpressed no
g
Pso T4 vs. Pso T1
diffexpressed DE no
P048442
h
Pso T1 vs. Pso baseline
diffexpressed DE no
P16615
Q14974
Q6ZRY4
B4DR80
i
Pso T4 vs. Pso baseline
diffexpressed no
j
AD T4 vs. AD T1
diffexpressed no
k
AD T1 vs. AD baseline
diffexpressed no up
Q9Y6I33
l
AD T4 vs. AD baseline
diffexpressed no up
Q9Y6I34
m
Pso baseline vs. AD baseline
diffexpressed no up
Q9Y6I36
Q68BL76
-log10 (adj.p-value)
logFC

Suppl. Fig 2: Volcano plots of differentially expressed proteins in total platelet proteomics reveal no distinct inflammatory subtype.

Volcano plot of total platelet proteins in psoriasis patients (Pso) at baseline **(a)**, after one month of systemic therapy (T1) **(b),** and four months of systemic therapy (T4) **(c)** *versus* total platelets in healthy controls. Volcano plot of total platelet proteins in atopic dermatitis patients (AD) at baseline **(d)**, after one month of systemic therapy (T1) **(e)**, and four months of systemic therapy (T4) **(f)** *versus* total platelets in healthy controls. Timecourse comparison of total platelet proteins of T4 versus T1 in pso **(g)** and AD **(j)** patients, T1 versus baseline in Pso **(h)** and AD **(k)** patients and T4 versus baseline in Pso **(i)** and AD **(l)** patients. Comparison of total platelet proteins in Pso versus AD patients at baseline **(m).** -log10 adjusted p-values versus log fold change. Diffexpressed – differentially expressed, DE differentially expressed. Data represents the analysis of cohort 1 (n = 10 controls, n = 27 psoriasis patients, n = 4 AD patients), with addition of two psoriasis patients (n = 29 total), one AD patient (n = 5 total) and 5 controls (n = 15 total). Source data are can be accessed under PRIDE (http://www.ebi.ac.uk/pride), project PXD057615.

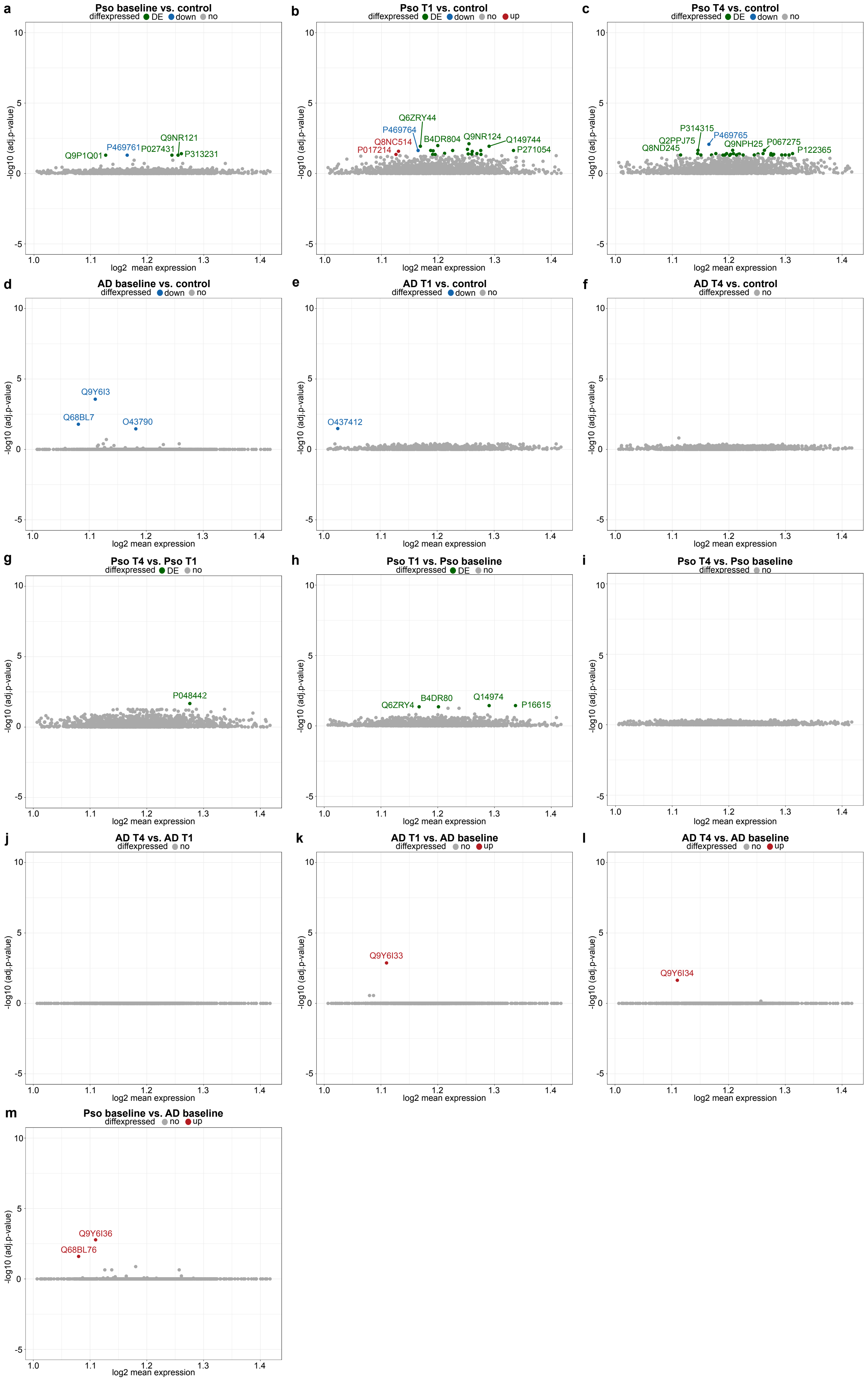
a
Pso baseline vs. control
diffexpressed DE down no
Q9NR121
P469761
P027431
Q9P1Q01
P313231
b
Pso T1 vs. control
diffexpressed DE down no up
Q6ZRY44
P469764
B4DR804
Q9NR124
Q8NC514
Q149744
P017214
P271054
c
Pso T4 vs. control
diffexpressed DE down no
P314315
P469765
Q2PPJ75
P067275
Q8ND245
Q9NPH25
P122365
d
AD baseline vs. control
diffexpressed down no
Q9Y6I3
Q68BL7
O43790
e
AD T1 vs. control
diffexpressed down no
O437412
f
AD T4 vs. control
diffexpressed no
g
Pso T4 vs. Pso T1
diffexpressed DE no
P048442
h
Pso T1 vs. Pso baseline
diffexpressed DE no
B4DR80
Q14974
Q6ZRY4
P16615
i
Pso T4 vs. Pso baseline
diffexpressed no
j
AD T4 vs. AD T1
diffexpressed no
k
AD T1 vs. AD baseline
diffexpressed no up
Q9Y6I33
l
AD T4 vs. AD baseline
diffexpressed no up
Q9Y6I34
m
Pso baseline vs. AD baseline
diffexpressed no up
Q9Y6I36
Q68BL76
-log10 (adj.p-value)
log2 mean expression

Suppl. Fig. 3: Volcano plots of differentially expressed proteins in total platelet proteomics reveal no distinct inflammatory subtype.

Adjusted p-value versus log2 mean expression of total platelet proteins in psoriasis patients (Pso) at baseline **(a)**, after one month of systemic therapy (T1) **(b),** and four months of systemic therapy (T4) **(c)** *versus* total platelets in healthy controls. Adjusted p-value versus log2 mean expression of total platelet proteins in atopic dermatitis patients (AD) at baseline **(d)**, after one month of systemic therapy (T1) **(e)**, and four months of systemic therapy (T4) **(f)** *versus* total platelets in healthy controls. Timecourse comparison of total platelet proteins of T4 versus T1 in Pso **(g)** and AD **(j)** patients, T1 versus baseline in Pso **(h)** and AD **(k)** patients and T4 versus baseline in Pso **(i)** and AD **(l)** patients. Comparison of total platelet proteins in Pso versus AD patients at baseline **(m).** log10 adjusted p-values versus log2 mean expression of total platelet proteins. Diffexpressed – differentially expressed, DE differentially expressed. Data represents the analysis of cohort 1 (n = 10 controls, n = 27 psoriasis patients, n = 4 AD patients), with addition of two psoriasis patients (n = 29 total), one AD patient (n = 5 total) and 5 controls (n = 15 total). Source data are can be accessed under PRIDE (http://www.ebi.ac.uk/pride), project PXD057615.

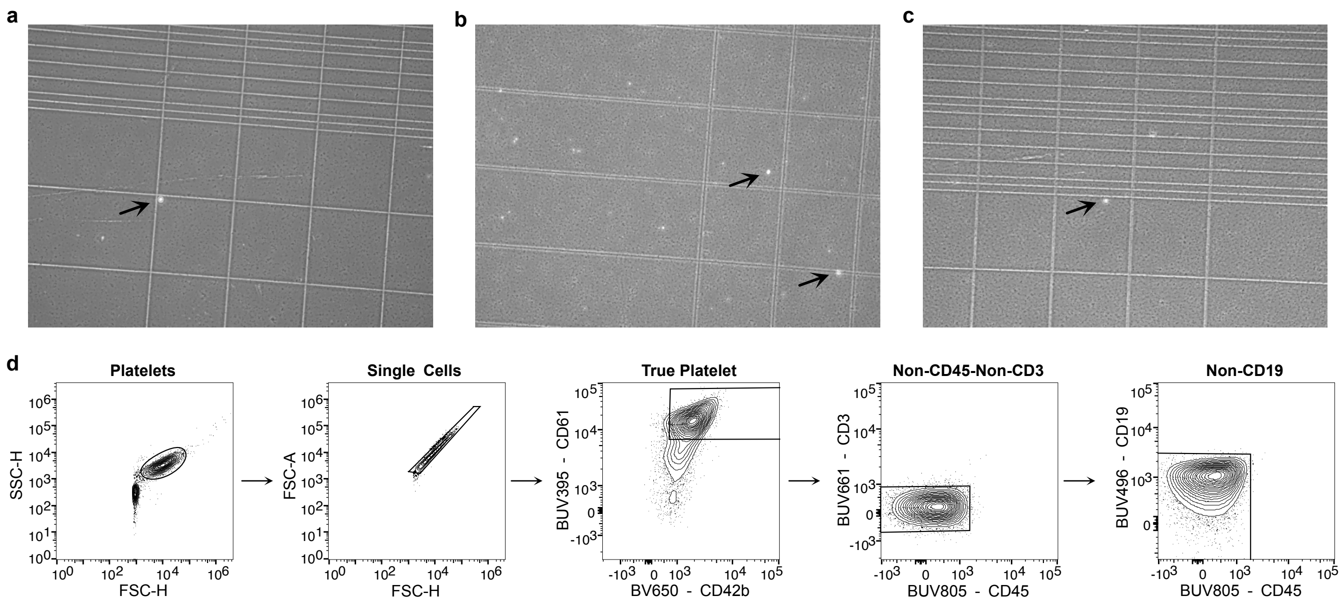

Suppl. Fig. 4: Platelet samples contain minor leukocyte contamination.

a-c. Representative images of three individual patient platelet pellets of 9 ml blood samples used for proteomic analyses. The platelet pellet was resuspended in 50 µl of Tyrode’s buffer, and 1 µl of the suspension diluted 1:50 in Tyrode’s buffer. Pictures were taken at original image x 100 magnification within a Neubauer chamber. Arrows indicate leukocytes. **d.** Gating strategy of high dimensional flow cytometry analysis with representative plots.

SUPPLEMENTARY TABLES

COHORT 1	PSORIASIS (n=27) n (%; ±SD)	AD (n=4) n (%; ±SD)	CONTROLS (n=10) n (%; ±SD)
**Male sex**	19 (70.37%)	1 (25.0%)	7 (70.0%)
**Age** [years]	36.56 (±13.61)	28.25 (±9.15)	29.20 (±3.76)
**BMI**	30.18 (±10.40)	26.67 (±5.93)	20.57 (±2.08)
**Psoriasis arthritis**	8 (29.63%)	0 (0%)	0 (0%)
**Art. hypertension**	2 (7.40%)	0 (0%)	0 (0%)
**DM type II**	2 (7.40%)	0 (0%)	0 (0%)
**Asthma**	4 (14.81%)	1 (25.0%)	0 (0%)
**RCA**	6 (22.22%)	3 (75.0%)	0 (0%)
**Smoking**	13 (48.15%)	0 (0%)	0 (0%)
**Pack Years**	6 (±9.86)	0 (0%)	0 (0%)
**PASI/EASI** Baseline	17.19 (±9.53)	19.93 (±6.30)	-
**PASI/EASI** T1	3.76 (±3.42)	5.47 (±6.89)	-
**PASI/EASI** T4	2.0 (±1.97)	5.4 (±3.93)	-
**Delta PASI/EASI** Baseline *vs.* T4	15.98 (±9.30)	13.67 (±6.22)	-
**IL-17 inhibitor**<br>Ixekizumab<br>Secukinumab<br>Brodalumab	14 (51.85%)<br>12 (44.44%)<br>1 (3.70%)<br>1 (3.70%)	-	-
**IL-23 inhibitor**<br>Risankizumab<br>Guselkumab<br>Tildrakizumab	7 (25.93%)<br>3 (11.11%)<br>3 (11.11%)<br>1 (3.70%)	-	-
**TNF inhibitor**<br>Adalimumab	2 (7.40%)<br>2 (7.40%)	-	-
**MTX**	2 (7.40%)	-	-
**IL-4/13 inhibitor**<br>Dupilumab	-	2 (50.0%)<br>2 (50.0%)	-
**JAK inhibitor**<br>Baricitinib	-	1 (25.0%)<br>1 (25.0%)	-

Suppl. Table 1: Overview over patient characteristics. Patients were followed prospectively (baseline, 1 month post systemic therapy (T1), 4 months post systemic therapy (T4)). Body mass index (BMI), arterial hypertension (art. hypertension), diabetes mellitus type II (DM type II), rhinoconjunctivitis allergica (RCA), psoriasis area and severity index (PASI), eczema area and severity index (EASI). Data are presented as absolute values (n) with per cent of total cohort (%), or mean (± standard deviation (SD)).

Marker	Clone	Fluorochrome	Company	Dilution
CD61	RUU-PLF712	**BUV395**	BD	100
CD19	SJ25C1	**BUV496**	BD	150
CD274	MIH1	**BUV563**	BD	200
CCR4	1G1	**BUV615-p**	BD	200
CD3	UCHT1	**BUV661**	BD	100
CD154	TRAP1	**BUV737**	BD	50
CD45	HI-30	**BUV805**	BD	100
CXCR4	12G5	**BV510**	Biolegend	75
CCR3	5E8	**BV605**	Biolegend	75
CD42b	HIP1	**BV650**	Biolegend	100
CD62P	AK4	**BV711**	Biolegend	75
CD49f	GoH3	**Super Bright 780**	ThermoScientific	100
CD282 (TLR2)	W15145C	**FITC**	Biolegend	50
CD66b	na	**BB790**	BD	50
TLR4	TF901	**PerCP-eFluor 710**	ThermoScientific	100
CXCR1	8F1/CXCR1	**PE-Cy5**	Biolegend	100
CXCR7	358426	**PE**	R&D	75
P2Y12		**PE-Dazzle**	Biolegend	50
GARP (LRRC32)	7B11	**Pe-Cy7**	Biolegend	50
Fc gamma RII/CD32		**APC**	R&D	50
CCR1	5F10B29	**APC-C7**	Biolegend	150
CD14	16D3	**Spark NIR**	Biolegend	250
TLR9	S16013D	**BV421**	Biolegend	50
TLR7	533707	**AF700**	R&D	50

Suppl. Table 2: List of antibodies, see material and methods section.

Replicate	Psi	Phi	Eigen 1	Eigen 2	%
1	1.7566**	0.2964**	2.16099**	0.90139	34.3
2	0.6518**	0.1805**	1.66782**	1.05991	29.3
3	1.4368**	0.268**	2.04434**	0.93271	34.9
4	2.5494**	0.357**	2.41**	0.79429	42.5
5	1.475**	0.2716**	2.0568**	0.93381	30.2
6	1.6425**	0.2866**	2.12077**	0.90643	33.1
7	0.8952**	0.2116**	1.82259**	0.9234	35.9
8	1.8752**	0.3062**	2.2045**	0.89559	50.8
9	1.698**	0.2914**	2.11942**	0.9918	26.3
10	1.1322**	0.2379**	1.91025**	1.00805	27.4
11	0.5831**	0.1708**	1.60362**	1.13362	38.4
12	1.4636**	0.2705**	2.05622**	0.92873	37.6
13	0.897**	0.2118**	1.82462**	0.93181	34.1
14	0.5179**	0.1609**	1.44423*	1.27387**	35.7
15	1.68**	0.2898**	2.1426**	0.87212	36.3
16	0.9247**	0.215**	1.8084**	1.05486	32.7
17	1.0558**	0.2298**	1.89549**	0.9424	30.3
18	2.1561**	0.3283**	2.29951**	0.80253	29.6
19	1.1321**	0.2379**	1.89461**	0.99236	34.9
20	1.7474**	0.2956**	2.15167**	0.9208	34.7
21	1.252**	0.2502**	1.90418**	1.16134	27.1
22	1.1164**	0.2363**	1.89864**	1.02223	38.8
23	0.823**	0.2029**	1.77792**	0.96507	28.3
24	2.3461**	0.3425**	2.34438**	0.86256	30.3
25	2.0667**	0.3215**	2.26309**	0.86893	45.2
26	1.2175**	0.2467**	1.93979**	0.97615	28.0
27	1.1915**	0.2441**	1.91009**	1.03797	38.0
28	1.4759**	0.2717**	2.04058**	1.01773	36.1
29	0.8241**	0.203**	1.78917**	0.9357	29.0
30	2.3987**	0.3463**	2.36265**	0.86908	32.8
31	1.1261**	0.2373**	1.93037**	0.89179	38.6
32	1.7562**	0.2963**	2.14773**	0.94424	37.9
33	1.4847**	0.2725**	2.04018**	0.99657	26.6
34	0.5223**	0.1616**	1.57732**	1.07762	42.7
35	1.3421**	0.259**	2.01685**	0.90325	31.1
36	1.1955**	0.2445**	1.96505**	0.86568	29.0
37	1.0792**	0.2323**	1.8568**	1.01959	33.8
38	1.1077**	0.2353**	1.90972**	0.96785	34.5
39	2.8435**	0.3771**	2.49904**	0.75457	38.2
40	0.7303**	0.1911**	1.72285**	0.98696	29.6
41	1.6305**	0.2855**	2.07245**	1.04891	27.2
42	0.6441**	0.1795**	1.65273**	1.06766	33.1

43	1.4749**	0.2716**	2.04767**	0.96497	37.2
44	1.7058**	0.292**	2.13416**	0.92282	34.2
45	1.4037**	0.2649**	2.03467**	0.90681	32.9
46	1.4766**	0.2717**	2.04257**	0.95821	27.5
47	1.5573**	0.279**	2.07584**	0.9722	36.8
48	1.4797**	0.272**	2.06738**	0.8985	34.9
49	1.2042**	0.2454**	1.96494**	0.87312	34.1
50	1.503**	0.2741**	2.07028**	0.89289	28.9
51	1.613**	0.284**	2.09448**	0.95085	33.3
52	1.1665**	0.2415**	1.93515**	0.964	34.5
53	0.7647**	0.1955**	1.68738**	1.14345	40.2
54	1.8534**	0.3044**	2.18591**	0.8952	35.6
55	1.0102**	0.2247**	1.84398**	0.95495	29.8
56	0.8625**	0.2077**	1.79551**	0.97757	35.8
57	1.9811**	0.3147**	2.24678**	0.82755	37.0
58	1.6841**	0.2902**	2.14086**	0.87196	33.8
59	0.792**	0.199**	1.74833**	1.03178	33.8
60	1.4243**	0.2669**	2.04735**	0.91107	32.0
61	1.3859**	0.2632**	1.99501**	0.96755	33.1
62	0.8807**	0.2098**	1.80412**	0.95929	27.9
63	1.1839**	0.2433**	1.92821**	1.01063	37.2
64	0.8494**	0.2061**	1.78421**	0.93734	38.4
65	1.1994**	0.2449**	1.92517**	0.96211	36.8
66	1.6311**	0.2856**	2.10007**	0.99289	31.5
67	0.9395**	0.2167**	1.8138**	1.01376	32.5
68	0.7874**	0.1984**	1.72588**	1.066	27.5
69	1.9529**	0.3125**	2.22526**	0.8859	29.9
70	0.9401**	0.2168**	1.80343**	1.06297	42.1
71	0.8723**	0.2088**	1.80828**	0.91913	28.5
72	0.7329**	0.1914**	1.72396**	0.99815	49.9
73	1.5339**	0.2769**	2.09411**	0.865	35.6
74	2.3306**	0.3414**	2.35845**	0.73767	37.0
75	0.5237**	0.1618**	1.60427**	1.01411	29.7
76	1.2726**	0.2523**	1.94732**	1.04398	39.6
77	1.3248**	0.2574**	1.95044**	1.09827	40.5
78	1.7651**	0.2971**	2.16927**	0.86422	33.8
79	1.5036**	0.2742**	2.04028**	1.01296	34.2
80	2.1099**	0.3248**	2.28981**	0.78751	29.4
81	1.5878**	0.2818**	2.06612**	0.98936	34.3
82	1.5097**	0.2747**	2.06615**	0.92991	37.9
83	1.354**	0.2602**	2.01829**	0.90858	35.4
84	1.1074**	0.2353**	1.88637**	1.04555	34.7
85	1.4095**	0.2655**	2.04207**	0,.85894	35.2

86	1.6526**	0.2875**	2.11452**	0.91514	35.2
87	0.796**	0.1995**	1.7559**	1.02061	35.2
88	1.0712**	0.2314**	1.86576**	1.0556	31.7
89	0.8113**	0.2014**	1.71731**	1.08323	36.5
90	2.3237**	0.3409**	2.34133**	0.85624	30.8
91	1.2225**	0.2472**	1.95543**	0.95391	35.9
92	0.8732**	0.2089**	1.77158**	1.05683	33.6
93	1.1008**	0.2346**	1.90837**	0.96633	42.3
94	1.0539**	0.2296**	1.8851**	0.93309	29.3
95	1.1311**	0.2378**	1.92281**	0.97094	36.4
96	2.6301**	0.3626**	2.42995**	0.82988	34.1
97	1.8293**	0.3024**	2.1918**	0.84039	35.8
98	1.7743**	0.2979**	2.14095**	0.98372	41.7
99	0.9537**	0.2184**	1.78809**	1.11594	28.3
100	1.6059**	0.2834**	2.11352**	0.91277	33.3
					34.3

Suppl. Table 3: PCATest related to Figure 1B, 100 repeats. PCAtest to evaluate the overall significance of the PC Analysis in Figure 1b. Taking PC axes 1 and 2 at Baseline, the contributions of each observed variable to the significant axes are based on permutation-based statistical tests. One-sided t-test, adjusted for multiple comparisons.

Uniprot	Gene	CD/alt. Name	Contrast	logFC	Ave. Expr.	t	P-Val	Adj. P Val
P29965	CD40LG	CD154	AD T0	-0.751	14.480	-1.547	0.127	0.997
			Pso T0	0.366	14.480	1.309	0.195	0.745
			AD T1	0.134	14.480	0.233	0.817	0.992
			AD T4	0.753	14.480	1.305	0.197	0.774
			Pso T1	0.316	14.480	1.162	0.250	0.631
			Pso T4	0.304	14.480	1.055	0.295	0.646
P31994	FCGR2B	CD32	n.d.	n.d.	n.d.	n.d.	n.d.	n.d.
P12318	FCGR2A		n.d.	n.d.	n.d.	n.d.	n.d.	n.d.
P31995	FCGR2C		n.d.	n.d.	n.d.	n.d.	n.d.	n.d.
O60603	TLR2	CD282	n.d.	n.d.	n.d.	n.d.	n.d.	n.d.
O00206	TLR4	CD284	n.d.	n.d.	n.d.	n.d.	n.d.	n.d.
Q9H244	P2RY12	P2Y12	AD T0	-0.128	14.829	-0.467	0.641	0.997
			Pso T0	-0.003	14.829	-0.023	0.982	0.999
			AD 1mo	-0.004	14.829	-0.012	0.991	0.998
			AD T4	-0.060	14.829	-0.201	0.841	0.980
			Pso T1	0.240	14.829	1.523	0.130	0.490
			Pso T4	-0.198	14.829	-1.258	0.211	0.556
Q14392	LRRC32	GARP	AD T0	0.072	17.092	0.369	0.712	0.997
			Pso T0	0.087	17.092	0.842	0.402	0.874
			AD T1	0.188	17.092	0.877	0.382	0.863
			AD T4	-0.092	17.092	-0.427	0.670	0.949
			Pso T1	0.151	17.092	1.346	0.181	0.563
			Pso T4	0.043	17.092	0.385	0.701	0.881

Suppl. Table 4: Total platelet-proteome analysis of the investigated cohort of patients with atopic dermatitis (AD) or psoriasis (Pso) in comparison to healthy controls does not show inflammation-specific signature of the investigated surface markers. Patients (AD/Pso) in comparison to healthy controls, stratified by marker and timepoint of investigation (baseline (T0), 1 month post systemic therapy (T1), 4 months post systemic therapy (T4)). Cluster of differentiation antigen (CD). Alternative name (alt. name). Average Expression (Ave. Expr.). logFC (log2 transformed expression value). Not detected (n.d.). Multiple testing with Benjamini and Hochberg (BH) adjusted p value (adj. P. Val). Differential expression was analyzed in the R package Limma using linear models and moderated two-sided t-tests. Resulting nominal p-values were adjusted using the Benjamini-Hochberg (BH) method to control the False Discovery Rate (FDR), with a significance threshold set at FDR ≤0.05.

Uniprot	Gene	CD/alt. Name	Contrast	logFC	Ave. Expr.	t	P-Val	Adj. P Val
P29965	CD40LG	CD154	Pso T1 *vs* Pso T0	-0.050	14.537	-0.218	0.828	0.957
			Pso T4 *vs* Pso T0	-0.061	14.537	-0.252	0.802	0.982
			Pso T4 *vs* Pso T1	-0.012	14.537	-0.049	0.961	0.989
			AD T1 *vs* AD T0	0.885	14.537	1.464	0.149	0.999
			AD T4 *vs* AD T0	1.504	14.537	2.487	0.016	1.000
			AD T4 *vs* AD T1	0.619	14.537	0.934	0.355	0.999
			Pso T0*vs* AD T0	1.117	14.537	2.679	0.010	0.991
P31994	FCGR2B	CD32	n.d.	n.d.	n.d.	n.d.	n.d.	n.d.
P12318	FCGR2A		n.d.	n.d.	n.d.	n.d.	n.d.	n.d.
P31995	FCGR2C		n.d.	n.d.	n.d.	n.d.	n.d.	n.d.
O60603	TLR2	CD282	n.d.	n.d.	n.d.	n.d.	n.d.	n.d.
O00206	TLR4	CD284	n.d.	n.d.	n.d.	n.d.	n.d.	n.d.
Q9H244	P2RY12	P2Y12	Pso T1 *vs* Pso T0	0.243	14.829	1.642	0.104	0.622
			Pso T4 *vs* Pso T0	-0.195	14.829	-1.315	0.192	0.763
			Pso T4 *vs* Pso T1	-0.438	14.829	-2.748	0.007	0.165
			AD T1 *vs* AD T0	0.124	14.829	0.335	0.738	0.999
			AD T4 *vs* AD T0	0.067	14.829	0.181	0.857	1.000
			AD T4 *vs* AD T1	-0.057	14.829	-0.146	0.884	0.999
			Pso T0*vs* AD T0	0.124	14.829	0.469	0.640	0.999
Q14392	LRRC32	GARP	Pso T1 vs Pso T0	0.064	17.111	0.589	0.557	0.885
			Pso T4 *vs* Pso T0	-0.044	17.111	-0.408	0.684	0.957
			Pso T4 *vs* Pso T1	-0.108	17.111	-0.926	0.357	0.731
			AD T1 *vs* AD T0	0.116	17.111	0.429	0.669	0.999
			AD T4 *vs* AD T0	-0.163	17.111	-0.605	0.547	1.000
			AD T4 *vs* AD T1	-0.279	17.111	-0.980	0.330	0.999
			Pso T0*vs* AD T0	0.015	17.111	0.080	0.937	0.999

Suppl. Table 5: Total platelet-proteome analysis of the investigated cohort of patients with atopic dermatitis (AD) or psoriasis (Pso) does not show inflammation-specific signature of the investigated surface markers within the different timepoints. Patients

(AD/Pso) stratified by marker and timepoint of investigation (baseline (T0), 1 month post systemic therapy (T1), 4 months post systemic therapy (T4)). Cluster of differentiation antigen (CD). Alternative name (alt. name). Average Expression (Ave. Expr.). logFC (log2 transformed expression value). Adjusted p value (adj. P. Val). Not detected (n.d.). Differential expression was analyzed in the R package Limma using linear models and moderated two-sided t-tests. Resulting nominal p-values were adjusted using the Benjamini-Hochberg (BH) method to control the False Discovery Rate (FDR), with a significance threshold set at FDR ≤0.05.

	Uniprot	Gene	Protein	log FC	adj. P Val
**Pso baseline**	P02743	APCS	amyloid P component, serum	0.562	0.050
	P31323	PRKAR2B	protein kinase cAMP-dependent type II regulatory subunit beta	0.401	0.039
	P46976	GYG1	glycogenin 1	-1.589	0.050
	Q9NR12	PDLIM7	PDZ and LIM domain 7	-0.285	0.050
	Q9P1Q0	VPS54	VPS54 subunit of GARP complex	-0.518	0.050
**Pso T1**	A0A2C9F2R7	FRYL	FRY like transcription coactivator	-0.660	0.023
	B4DR80	IGLV6-57	immunoglobulin lambda variable 6-57	0.531	0.010
	P01721	RPN2	ribophorin II	1.149	0.044
	P04844	STK24	serine/threonine kinase 24	0.216	0.023
	P27105	STOM	stomatin	-0.258	0.023
	P31323	PRKAR2B	protein kinase cAMP-dependent type II regulatory subunit beta	0.379	0.027
	P46976	GYG1	glycogenin 1	-2.554	0.023
	P49411	CLINT1	clathrin interactor 1	-0.304	0.044
	P52306	KPNB1	karyopherin subunit beta 1	0.206	0.019
	Q05209	NEK7	NIMA related kinase 7	-0.369	0.038
	Q13464	PAAF1	proteasomal ATPase associated factor 1	-0.331	0.042
	Q14677	PDLIM5	PDZ and LIM domain 5	-0.496	0.037
	Q14974	PITPNM2	phosphatidylinositol transfer protein membrane associated 2	0.216	0.012
	Q6ZRY4	PTPN12	protein tyrosine phosphatase non-receptor type 12	-0.592	0.012
	Q8NC51	RAP1GDS 1	Rap1 GTPase-GDP dissociation stimulator 1	3.004	0.026
	Q8TDX7	RBPMS2	RNA binding protein. mRNA processing factor 2	-0.325	0.024
	Q8WWI5	ROCK1	Rho associated coiled-coil containing protein kinase 1	0.290	0.041
	Q96HC4	SERBP1	SERPINE1 mRNA binding protein 1	-0.309	0.023
	Q9BRP4	SLC44A1	solute carrier family 44 member 1	0.386	0.045
	Q9BZ72	TUFM	Tu translation elongation factor. mitochondrial	-0.343	0.044
	Q9NR12	PDLIM7	PDZ and LIM domain 7	-0.375	0.008
**Pso T4**	O15111	APOE	apolipoprotein E	-0.355	0.038
	P01703	CHUK	component of inhibitor of nuclear factor kappa B kinase complex	0.882	0.046
	P02649	IGLV1-40	immunoglobulin lambda variable 1-40	0.655	0.041
	P02743	APCS	amyloid P component. serum	0.527	0.048
	P02763	A1BG	alpha-1-B glycoprotein	0.739	0.048
	P04217	APOA4	apolipoprotein A4	0.555	0.048
	P05141	CFP	complement factor properdin	-0.355	0.038

	P06727	DARS1	aspartyl-tRNA synthetase 1	0.880	0.022
	P10646	ORM1	orosomucoid 1	0.469	0.048
	P12236	SLC25A5	solute carrier family 25 member 5	-0.319	0.039
	P14868	SLC25A6	solute carrier family 25 member 6	-0.219	0.048
	P27918	TFPI	tissue factor pathway inhibitor	0.611	0.048
	P31323	PRKAR2B	protein kinase cAMP-dependent type II regulatory subunit beta	0.378	0.039
	P31431	SDC4	syndecan 4	0.844	0.022
	P36955	SERPINF1	serpin family F member 1	0.442	0.049
	P46976	GYG1	glycogenin 1	-2.587	0.008
	P50570	AIMP2	aminoacyl tRNA synthetase complex interacting multifunctional protein 2	-0.142	0.048
	P51884	AP1B1	adaptor related protein complex 1 subunit beta 1	0.588	0.041
	P53618	AP3M1	adaptor related protein complex 3 subunit mu 1	-0.191	0.049
	Q10567	ATP6V0A2	ATPase H+ transporting V0 subunit a2	-0.186	0.048
	Q13155	CD2AP	CD2 associated protein	-0.448	0.039
	Q14696	COPB1	COPI coat complex subunit beta 1	0.456	0.048
	Q16610	DIS3L2	DIS3 like 3'-5' exoribonuclease 2	0.367	0.043
	Q2PPJ7	DNAJC11	DnaJ heat shock protein family (Hsp40) member C11	-0.580	0.039
	Q8IYB7	DNM2	dynamin 2	-0.341	0.048
	Q8N1B4	ECM1	extracellular matrix protein 1	-0.266	0.043
	Q8ND24	FAM120A	family with sequence similarity 120A	-0.558	0.048
	Q9BZH6	IRAK3	interleukin 1 receptor associated kinase 3	-0.281	0.039
	Q9NPH2	ISYNA1	inositol-3-phosphate synthase 1	-0.429	0.022
	Q9NVH1	LUM	lumican	-0.283	0.048
	Q9NZB2	MESD	mesoderm development LRP chaperone	-0.355	0.049
	Q9Y2T2	RALGAPA2	Ral GTPase activating protein catalytic subunit alpha 2	-0.290	0.039
	Q9Y487	RNF214	ring finger protein 214	-0.231	0.048
	Q9Y5K6	VPS52	VPS52 subunit of GARP complex	0.521	0.049
	Q9Y616	WDR11	WD repeat domain 11	-0.421	0.048

Suppl. Table 6: Differentially expressed proteins in comparison to healthy controls within the psoriasis (Pso) cohort, stratified by timepoint of investigation (baseline, 1 month post systemic therapy (T1), 4 months post systemic therapy (T4)). Differential expression was analyzed in the R package Limma using linear models and moderated two-sided t-tests. Resulting nominal

p-values were adjusted using the Benjamini-Hochberg (BH) method to control the False Discovery Rate (FDR), with a significance threshold set at FDR ≤0.05.

	Uniprot	Gene	Protein	log FC	adj. P Val
**AD baseline**	O43790	KRT86	keratin 86	-6.500	0.035
	Q68BL7	OLFML2A	olfactomedin like 2A	-3.638	0.016
	Q9Y6I3	EPN1	epsin 1	-4.031	0.000
**AD T1**	O43741	PRKAB2	protein kinase AMP-activated non-catalytic subunit beta 2	-5.363	0.033
**AD T4**	n.d.	n.d.	n.d.	n.d.	n.d.

Suppl. Table 7: Differentially expressed proteins in comparison to healthy controls within the atopic dermatitis (AD) cohort, stratified by timepoint of investigation (baseline, 1 month post systemic therapy (T1), 4 months post systemic therapy (T4)). Not detected (n.d.). Differential expression was analyzed in the R package Limma using linear models and moderated two-sided t-tests. Resulting nominal p-values were adjusted using the Benjamini-Hochberg (BH) method to control the False Discovery Rate (FDR), with a significance threshold set at FDR ≤0.05.

COHORT 2	PSORIASIS (n=6) n (%; ±SD)	AD (n=10) n (%; ±SD)	CONTROLS (n=6) n (%; ±SD)
**Male sex**	2 (33.33%)	5 (50.0%)	4 (66.67%)
**Age** [years]	35.50 (±11.98)	37.90 (±13.07)	36.0 (±6.81)
**BMI**	30.99 (±6.84)	26.25 (±5.88)	22.66 (±2.16)
**Psoriasis Arthritis**	1 (16.67%)	0 (0%)	0 (0%)
**Art. Hypertension**	0 (0%)	1 (10.0%)	0 (0%)
**DM type II**	0 (0%)	0 (0%)	0 (0%)
**Asthma**	0 (0%)	5 (50.0%)	0 (0%)
**RCA**	0 (0%)	9 (90.0%)	0 (0%)
**Smoking**	2 (33.33%)	1 (10.0%)	0 (0%)
**Pack Years**	6.5 (±12.80)	2 (n.a.)	0 (0%)
**PASI/EASI** Baseline	14.0 (±3.86)	18.38 (±11.66)	-
**PASI/EASI** T1	3.50 (±3.11)	12.07 (±11.22)	-
**PASI/EASI** T4	1.93 (±2.44)	9.61 (±11.41)	-
**Delta PASI/EASI** Baseline *vs*. T4	12.07 (±4.75)	8.733 (±8.28)	-
**IL-17 inhibitor**<br>Ixekizumab	2 (33.33%)<br>2 (33.33%)	-	-
**IL-(12/)23 inhibitor**<br>Guselkumab<br>Ustekinumab	2 (33.33%)<br>1 (16.67%)<br>1 (16.67%)	-	-
**TNF inhibitor**<br>Adalimumab	1 (16.67%)<br>1 (16.67%)	-	-
**DMF**	1 (16.67%)	-	-
**IL-4/13 inhibitor**<br>Dupilumab<br>Tralokinumab	-	6 (60.0%)<br>4 (40.0%)<br>2 (20.0%)	-
**JAK inhibitor**<br>Baricitinib<br>Upadacitinib	-	4 (40.0%)<br>2 (20.0%)<br>2 (20.0%)	-

Suppl. Table 8: Overview over patient characteristics, validation cohort 2. Patients were followed prospectively (baseline, 1 month post systemic therapy (T1), 4 months post systemic therapy (T4)). Body mass index (BMI), arterial hypertension (art. hypertension), diabetes mellitus type II (DM type II), rhinoconjunctivitis allergica (RCA), psoriasis area and severity index (PASI), eczema area and severity index (EASI). Data are presented as absolute values (n) with per cent of total cohort (%), or mean (± standard deviation (SD)).

COHORT 3	PSORIASIS (n=21)<br>n (%; ±SD)	AD (n=9)<br>n (%; ±SD)	CONTROLS (n=8)<br>n (%; ±SD)
**Male sex**	14 (66.67%)	5 (50.55%)	3 (37.5%)
**Age** [years]	43.81 (±15.07)	41.22 (±12.26)	35.63 (±7.09)
**BMI**	30.00 (±6.11)	27.58 (±3.81)	27.23 (±7.59)
**Psoriasis Arthritis**	3 (14.29%)	0 (0%)	0 (0%)
**Art. Hypertension**	6 (28.57%)	1 (11.11%)	1 (12.5%)
**DM type II**	3 (14.29%)	0 (0%)	0 (0%)
**Asthma**	3 (14.29%)	4 (44.44%)	0 (0%)
**RCA**	6 (28.57%)	7 (77.78%)	0 (0%)
**Smoking**	6 (28.57%)	4 (44.44%)	0 (0%)
**Pack Years**	4.48 (±11.62)	8.28 (±15.97)	0 (0%)
**PASI/EASI**<br>Baseline	16.60 (±6.11)	21.17 (±11.02)	-
**PASI/EASI**<br>T1	5.23 (±1.79)	4.22 (±4.02)	-
**PASI/EASI**<br>T4	1.76 (±0.88)	3.55 (±2.60)	-
**Delta PASI/EASI**<br>Baseline *vs.* T4	12.94 (±5.08)	28.07 (±11.08)	-
**IL-17 inhibitor**<br>Secukinumab	2 (9.52%)<br>2 (9.52%)	-	-
**IL-23 inhibitor**<br>Risankizumab<br>Tildrakizumab<br>Guselkumab	7 (33.33%)<br>4 (19.05%)<br>2 (9.52%)<br>1 (4.76%)	-	-
**TNF inhibitor**<br>Adalimumab	3 (14.29%)<br>3 (14.29%)	-	-
**IL-4/13 inhibitor**<br>Dupilumab	-	4 (44.44%)<br>4 (44.44%)	-
**JAK inhibitor**<br>Upadacitinib	-	1 (11.11%)<br>1 (11.11%)	-

Suppl. Table 9: Overview over patient characteristics, validation cohort 3. Patients were followed prospectively (baseline, 1 month post systemic therapy (T1), 4 months post systemic therapy (T4)). Body mass index (BMI), arterial hypertension (art. hypertension), diabetes mellitus type II (DM type II), rhinoconjunctivitis allergica (RCA), psoriasis area and severity index (PASI), eczema area and severity index (EASI). Data are presented as absolute values (n) with per cent of total cohort (%), or mean (± standard deviation (SD)).
